# Detection of High-Risk Human Papillomavirus in Bladder Cancer: An Exploratory Study from a UK-Based Population

**DOI:** 10.3390/biomedicines13071548

**Published:** 2025-06-25

**Authors:** Mohammed Yahya Ahmed, Muharrem Okan Cakir, Sarbjinder Sandhu, G. Hossein Ashrafi

**Affiliations:** 1 School of Life Science, Pharmacy and Chemistry, Kingston University London, London KT1 2EE, UK; mohammedyahya1987@hotmail.com (M.Y.A.); m.okan@kingston.ac.uk (M.O.C.); 2 Kingston Hospital-Department of Urology and Surgery, Kingston upon Thames, London KT2 7QB, UK; sarb.sandhu@nhs.net

**Keywords:** human papillomavirus, HPV, high-risk HPV, bladder cancer, human papillomavirus, PCR, immunohistochemistry, oncoprotein E7

## Abstract

**Background/Objectives**: Human papillomavirus (HPV) is a prevalent sexually transmitted infection globally and is linked to the development of various cancers. While several international studies have investigated the incidence of high-risk HPV (HR-HPV) in bladder cancers, no such research has been conducted within the UK. Conflicting results in previous studies leave uncertainty regarding the role of HR-HPV in bladder cancer. This study aimed to assess the presence of HR-HPV DNA in bladder cancer specimens from the UK. **Methods**: A total of 55 fresh bladder specimens, including 4 benign and 51 malignant samples, were analysed using polymerase chain reaction (PCR) and Sanger sequencing to detect 12 HR-HPV types. Immunohistochemistry (IHC) was used to confirm the expression of the HPV E7 protein in HR-HPV-positive samples. **Results**: HR-HPV DNA was detected in 33% of bladder cancer specimens, with HPV16, HPV35, and HPV52 being the most prevalent types. None of the benign samples tested positive for HR-HPV. IHC confirmed HPV E7 protein expression in 81% of HR-HPV DNA-positive cancer samples. **Conclusions**: The findings suggest that HR-HPV may play a role in a subset of bladder cancers in the UK. The absence of HR-HPV in benign bladder specimens supports its potential involvement in cancer progression. Further research is needed to clarify the mechanistic role of HR-HPV in bladder cancer development.

## 1. Introduction

Bladder cancer is the sixth most common cancer worldwide with over 440,000 newly diagnosed cases annually and 160,000 fatalities [1]. The estimated number of new bladder cancer cases within the UK population alone is 10,200 with 5400 expected deaths annually, a number which unfortunately continues to rise steadily [2].

Bladder cancer was the first cancer to be associated with industrialisation due to the increased cancer cases amongst dye workers who worked with aromatic amines. Other risk factors for bladder cancer are known to include old age, cigarette smoking, male gender, arsenic in drinking water, and infection of the bladder [3,4,5].

Infectious agents, including high-risk Human Papillomaviruses (HR-HPVs), are recognised carcinogens in several cancer types. HPVs are small, non-enveloped viruses that infect epithelial surfaces such as the skin and genital areas through sexual or skin-to-skin contact, leading to hyper-proliferative lesions. There are over 200 identified HPV types, categorised as low-risk or high-risk based on their association with cancer development. While low-risk types cause benign lesions, high-risk types are strongly linked to cancer, particularly cervical cancer, which is attributed to HR-HPV in approximately 95% of cases [6,7].

Long-term persistence of HPV is critical for malignancy development, as it evades immune surveillance through the action of oncoproteins like E6 and E7. These oncoproteins disrupt key components of the immune system, such as MHC Class I and tumour suppressor proteins like p53 and Rb. This immune evasion allows HPV to persist and drive the progression of cancers such as cervical, vaginal, penile, anal, and oropharyngeal cancers [7,8,9,10,11,12]. Additionally, recent studies, including our own, have identified HR-HPV types in breast and prostate cancer tissues, supporting further investigation into HPV’s potential role in other cancers [13,14].

The relationship between bladder cancers and HPV types 16 and 18 as possible carcinogens was first proposed by Kitamura et al. (1988), who reported one HPV16-positive case among ten bladder tumours using Southern blot analysis [15]. Subsequently, Li et al. (2011) further supported this hypothesis, suggesting that high-risk HPV may contribute to bladder carcinogenesis [16]. However, several studies since then have failed to establish a definitive association between HPV and bladder cancer. For instance, Schmid et al. (2015) did not detect any HPV DNA in 109 bladder cancer samples [17].

While some studies have suggested the urethra as a potential reservoir for HPV [18], Griffith and Mellon (2000) found that HPV was present in 6% of urethral swabs, compared to 29% in bladder tissue, leading some authors to propose a link between HPV and bladder cancer [19]. However, the results have been highly controversial, with no conclusive evidence confirming a direct link between chronic HPV infection and bladder cancer. A recent meta-analysis by Khatami et al. (2022) [20] further explored the prevalence of HPV in bladder cancer, finding a pooled HPV prevalence of 14.3%. Despite this, no significant association was found between HPV and bladder cancer, underscoring the need for further case-control studies to better understand this potential connection [20]. Given the broad interest in HR-HPV and its possible role in bladder cancer, it is critical to investigate the prevalence of various other HR-HPV types in bladder cancer, beyond just HPV16 and HPV18, to clarify their involvement on a global scale.

To the best of our knowledge, no studies have been conducted in the UK to investigate the role of HR-HPV infection in bladder carcinogenesis, despite its significant medical importance and high incidence worldwide. Therefore, the aim of this study was to assess the presence and expression of 12 HR-HPV types in bladder tissue biopsies obtained from both benign and malignant cases in the UK, using highly sensitive molecular analysis techniques.

## 2. Materials and Methods

### 2.1. Recruitment of Patients and Bladder Tissue Specimen Collection

The study protocol was conducted in accordance with the Declaration of Helsinki and approved by the Ethics Committee of the Health Research Authority NHS (NRES Committee East Midlands—Leicester Central, UK; REC reference: 17/EM/0393). All procedures followed approved ethical guidelines and regulations. Following written informed consent, a total of 55 fresh bladder tissue specimens (51 malignant and 4 benign) were aseptically collected by a single surgical team over a two-year period. Tissues were immediately preserved in AllProtect reagent (Qiagen, Hilden, Germany) to stabilise DNA, RNA, and proteins. All specimens underwent histopathological evaluations at the Kingston Hospital Histopathology Department, London, UK.

### 2.2. DNA Extraction and Purification

To avoid cross-contamination during tissue handling, strict aseptic techniques were applied using disposable items such as gloves, sterile surgical blades, and tubes. Total cellular DNA, RNA, and proteins were extracted simultaneously from bladder tissue specimens using the GenElute RNA/DNA/Protein Purification Plus Kit (Sigma-Aldrich, St. Louis, MO, USA) according to the manufacturer’s instructions. Briefly, approximately 20–30 mg of each bladder tissue specimen was accurately weighed and homogenised in lysis buffer using a TissueLyser^®^ (Qiagen) and QIAshredder spin columns (Qiagen). Homogenised lysates were transferred to genomic DNA purification columns to selectively bind genomic DNA, followed by washing and elution using the provided elution buffer. The concentration and purity of extracted nucleic acids were assessed using a NanoVue Plus spectrophotometer (GE Life Sciences, Chicago, IL, USA).

### 2.3. Detection and Genotyping of HPV DNA

To detect and genotype 12 high-risk HPV (HR-HPV) subtypes, polymerase chain reaction (PCR) analysis was performed using the HPV-HCR Genotype-Eph kit (AmpliSens, Bratislava, Slovakia) on purified DNA from bladder tissue specimens. To minimise contamination risks, DNA extraction and PCR assays were conducted in separate laboratories under stringent aseptic conditions. Multiplex PCR was performed by simultaneously amplifying four HPV target regions within each tube, enabling the detection of the following HR-HPV groups: HPV types 16/31/33/35 (tube 1), HPV 18/39/45/59 (tube 2), and HPV 52/56/58/66 (tube 3). This methodology permitted identification of single HPV infections as well as co-infections. PCR reactions for each sample were performed in triplicate to validate data reliability. Amplification of the β-globin gene (723 bp fragment) served as an internal control for DNA integrity in all samples. To allow for semi-quantitative assessment, an internal standard consisting of synthetic DNA (10^3^ copies/µL) was added to each PCR reaction. This enabled relative comparison across samples by normalising for amplification efficiency and template variability.

Amplified PCR products and HPV type-specific positive controls were analysed by electrophoresis on 3% (*w*/*v*) agarose gels stained with SYBR Safe (Invitrogen, Carlsbad, CA, USA). The gel was visualised and documented under ultraviolet illumination using a Gel Doc XR + System (Bio-Rad, Hercules, CA, USA).

### 2.4. HPV DNA Sequencing

To confirm the presence and genotype accuracy of HPV DNA, PCR products from HPV-positive bladder cancer samples were purified using a QIAquick PCR Purification Kit (Qiagen) and subsequently subjected to direct sequencing using an Applied Biosystems 3730xL DNA Analyzer (Thermo Fisher Scientific, Foster City, CA, USA). Sequencing results were analysed and confirmed using the NCBI BLAST database (version 5) for HPV sequence validation.

### 2.5. Immunohistochemistry

To confirm HPV gene expression at the protein level, HPV DNA-positive bladder cancer tissue specimens underwent immunohistochemical analysis for HPV E7 protein using an anti-HPV E7 monoclonal antibody (Cervimax, Valdospan GmbH, Tulln an der Donau, Austria) that targets a broad range of HR-HPV types. IHC staining was performed using an automated BenchMark Ultra system (VENTANA, Roche Diagnostics, Basel, Switzerland) according to the manufacturer’s protocol. The HPV E7 monoclonal antibody was applied at a dilution of 1:100 and incubated at 36 °C for one hour. Cervical intraepithelial neoplasia grade III (CIN III) tissue blocks served as positive controls, while slides incubated without primary antibody served as negative controls. The staining intensity and positivity were quantified using ImageJ software (version 1.54p, NIH, Bethesda, MD, USA) with the IHC Analyzer plugin. Staining intensity was scored as negative (no staining detected), positive (visible only at high magnification), or strongly positive (visible clearly at low magnification).

## 3. Results

### 3.1. Clinicopathological Characteristics of the Patients

The clinical and pathological characteristics of the bladder specimens analysed in this study are summarised in Table 1. All pathological diagnoses were confirmed by the histopathology department of Kingston Hospital, London, UK, based on comprehensive histological evaluation of biopsy specimens.

A total of 55 fresh bladder tissue samples (47 male, 8 female) were collected from patients undergoing evaluation for suspected bladder cancer at the Urology Department of Kingston Hospital. Patient age ranged from 45 to 96 years at the time of biopsy, with the following age distribution: 6 (10.9%) patients younger than 60 years, 10 (18.2%) aged 61–70 years, 18 (32.7%) aged 71–80 years, and 21 (38.2%) patients older than 81 years.

Histopathological examination identified 51 malignant bladder cancer samples, including 49 (89.1%) transitional cell carcinoma (urothelial carcinoma) and 2 (3.6%) squamous cell carcinoma. The remaining 4 (7.3%) samples were benign bladder lesions.

### 3.2. Detection of HR-HPV DNA in Bladder Cancer Specimens

Extracted DNA from all 55 bladder tissue specimens was analysed for the presence of 12 high-risk HPV (HR-HPV) types (HPV-16, 18, 31, 33, 35, 39, 45, 52, 56, 58, 59, and 66) using multiplex PCR. Each PCR experiment was conducted in triplicate to ensure data reliability and reproducibility. HPV-positive and negative controls were included in every run. Additionally, amplification of a 723 bp fragment of the β-globin gene served as an internal control to confirm DNA integrity and suitability of each sample for PCR analysis.

**Figure 1:**
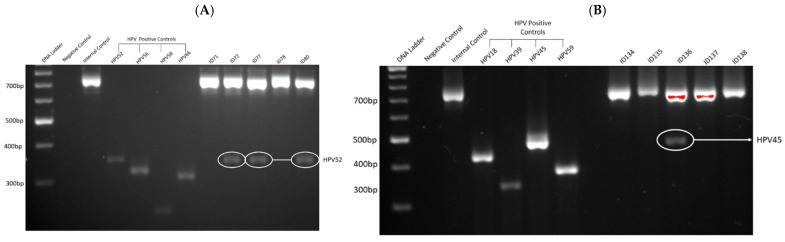
A representative gel electrophoresis pattern of 12 high-risk HPV types by genotype specific primers amplification. (**A**) Gel electrophoresis pattern of high-risk HPV types (52, 56, 58, and 66), Loading Marker = DNA ladder 100 bp plus (100 bp–3000 bp), HPV 52/66 = Positive Control DNA HPV types, 52 (360 bp), 56 (325 bp), 58 (240 bp), and 66 (304 bp), respectively, ID 71 and 78 = HPV Negative clinical sample, ID 72. 77 and 80 = HPV Positive clinical sample, Positive Control (PC+) = Internal control; human DNA (β -globin 723 bp). (**B**) Gel electrophoresis pattern of high-risk HPV types (18, 39, 45, and 59), HPV 18/59 = Positive Control DNA, HPV types 18 (425 bp), 39 (340 bp), 45 (340 bp), and 59 (395 bp) respectively, ID 134, 135, 137, and 138 = HPV Negative clinical sample, ID 136 = HPV Positive clinical sample. An internal standard (10^3^ copies/µL synthetic DNA control) was spiked into each reaction to enable semi-quantitative normalisation of target gene amplification.

The β-globin gene was successfully amplified in all 55 cases, confirming that DNA extraction and amplification were efficient. As shown in Figure 1, gel electrophoresis demonstrated clear amplification products for HPV-positive controls, with no amplification observed in negative controls, indicating the absence of contamination and validating the efficiency and specificity of the PCR assay. Figure 1A,B illustrate representative examples of successful PCR amplification for HR-HPV in bladder cancer specimens. Regarding HPV prevalence, HR-HPV DNA was detected in 33% of malignant bladder specimens, with HPV16, HPV35, and HPV52 being the most commonly identified genotypes. No HPV DNA was detected in squamous cell carcinoma (SCC) and benign bladder tissue samples.

### 3.3. Sanger Sequencing Results

**Figure 2:**
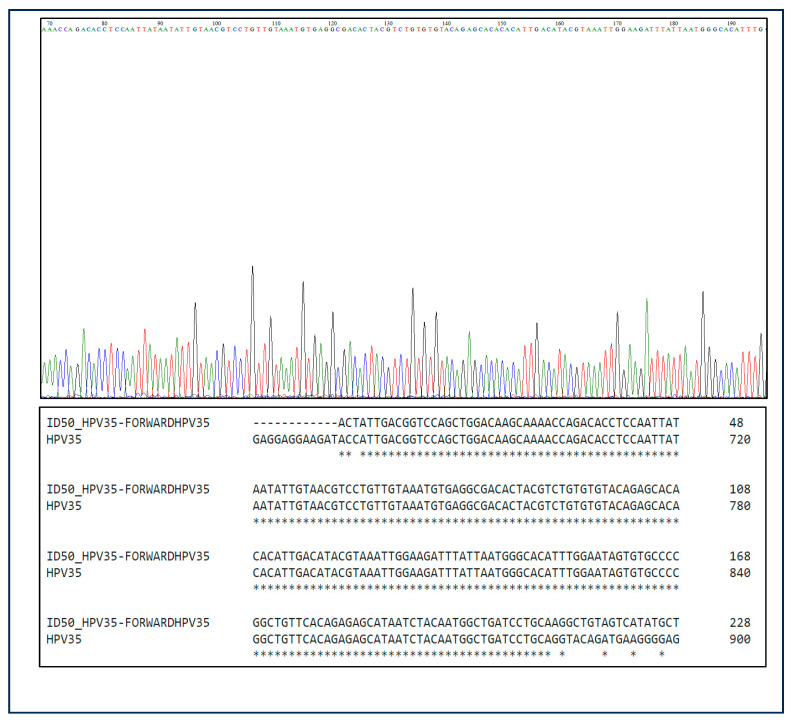
Sanger sequencing analysis further validates and confirms the PCR results. A representative Sanger sequencing result (Sample ID 50) shows 90% concordance for HPV35. This applies to all PCR results.

All HR-HPV-positive bladder samples identified by PCR were subjected to Sanger sequencing to confirm the PCR-based genotyping results. A total of 17 PCR-amplified HR-HPV-positive samples were sequenced, and all 17 samples were confirmed to be positive for HPV DNA, showing more than 90% concordance with the PCR results. In contrast, selected HPV-negative samples were also subjected to sequencing as controls. Out of the 38 PCR-amplified HPV-negative samples, 37 samples were confirmed as negative by sequencing, while one sample showed a faint HPV sequence that was not considered significant, confirming the specificity and accuracy of the PCR assay representative chromatograms from Sanger sequencing analysis that are presented in Figure 2.

### 3.4. Prevalence and Distribution of HR-HPV Genotypes in Bladder Cancer

Among the 51 malignant bladder samples, HR-HPV DNA was detected in 17 cases (33.3%). The most identified genotypes were HPV16, HPV35, and HPV52, each detected in 5 out of 49 (10.2%) transitional cell carcinoma (TCC) samples (Table 2). Other detected genotypes included HPV33, HPV39, HPV45, HPV59, and HPV66, though at lower frequencies. HPV18, HPV31, HPV56, and HPV58 were not detected in any sample. Table 2 details the genotype-specific distribution of HR-HPVs by tumour grade. Among the 51 malignant cases, HPV was detected in 8/16 (50%) of Grade 1 tumours, 2/14 (14.3%) of Grade 2 tumours, and 7/19 (36.8%) of Grade 3 tumours. Although HPV prevalence appeared higher in Grade 1 tumours (8/16, 50%) than in Grade 2 and 3 combined (9/33, 27.3%), the difference was not statistically significant (*p* > 0.05, Fisher’s exact test).

### 3.5. Frequency and Grade-Based Analysis of HPV Co-Infection

Of the 17 HR-HPV-positive TCC cases, 6 samples (12.2%) demonstrated co-infection with multiple HR-HPV genotypes (Table 3). Co-infections were more frequently observed in Grade 1 tumours (3 out of 16, or 19%) compared to Grade 3 (2/19, 11%) and Grade 2 (1/14, 7%). This may suggest that co-infections can occur early in tumour development, although due to the limited number of cases, no statistically significant association could be drawn between co-infection status and tumour grade. Table 3 outlines the number and percentage of co-infections stratified by tumour grade, showing that HPV co-infection is not restricted to higher-grade malignancies.

### 3.6. HPV Co-Infection Patterns and Combinatorial Genotypes

To understand the types of co-infection, Table 4 presents the specific genotype combinations detected in co-infected bladder cancer tissues. Double HPV infections were observed in four cases, including combinations such as HPV16–HPV39, HPV35–HPV66, HPV52–HPV66, and HPV45–HPV59. Two samples showed triple infections: HPV16–HPV33–HPV52 and HPV35–HPV45–HPV59.

### 3.7. The Expression of HPV Protein in Samples Positive for HPV

Immunohistochemical (IHC) analysis was performed to investigate HPV E7 oncoprotein expression in all 55 bladder specimens, including both benign and malignant samples. HPV-E7 protein expression was detected in 13 of these 17 HPV positive bladder cancer samples (81%). In contrast, no E7 expression was detected in HPV DNA-negative samples. Additionally, four HPV DNA-positive samples did not exhibit detectable HPV E7 expression.

**Figure 3:**
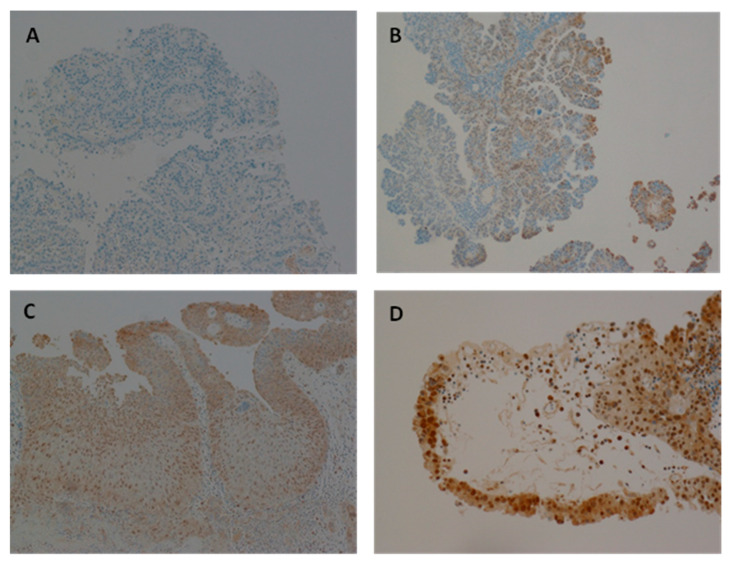
Expression of HPV oncoprotein E7 analysed using immunohistochemistry. (**A**) Representative of bladder cancer with HPV showing negative HPV expression. (**B**) Representative of HPV positive bladder cancer sample with slightly positive HPV expression. (**C**,**D**) Representative of HPV infected bladder cancer sample with moderate and absolute HPV expression, respectively.

Representative immunohistochemical staining patterns for HPV E7 expression in bladder cancer tissues are shown in Figure 3. HPV E7 expression was absent in the HPV DNA-negative bladder cancer sample (Figure 3A), serving as a negative control. In contrast, Figure 3B illustrates an HPV DNA-positive bladder cancer sample exhibiting weak E7 expression, while Figure 3C,D depict HPV DNA-positive samples with moderate and strong E7 expression, respectively.

## 4. Discussion

Human papillomavirus (HPV) is one of the most prevalent sexually transmitted infections globally, with most sexually active individuals acquiring it during their lifetime. While HPV is well recognised as a causal factor in cervical and several anogenital cancers [6,11,12,13], its role in bladder carcinogenesis remains a subject of debate. Bladder cancer is the sixth most common cancer worldwide, with a significant and steadily increasing incidence in both men and women [1,2]. However, the presence and pathogenic role of high-risk HPV (HR-HPV) in bladder cancer, especially within the UK population, has not been adequately investigated.

In this study, HR-HPV DNA was detected in 17 out of 51 malignant bladder samples (33.3%), using sensitive multiplex PCR and confirmed through Sanger sequencing. Among the 12 HR-HPV types analysed, HPV16, HPV35, and HPV52 were the most prevalent, each found in 5 out of 49 transitional cell carcinoma (TCC) samples (Table 2). Other genotypes detected at lower frequencies included HPV33, HPV39, HPV45, HPV59, and HPV66, whereas HPV18, HPV31, HPV56, and HPV58 were not identified in any of the specimens. The detection of diverse HR-HPV types, beyond the commonly studied HPV16 and HPV18, underscores the importance of broader genotyping in HPV-related cancer research [6,14].

These findings partially align with those of Li et al. (2011), who also identified HPV16 as the most prevalent genotype in bladder cancers but reported HPV18 as less frequently involved [15]. In contrast, Jørgensen and Jensen (2020) and Yan et al. (2021) found HPV18, HPV31, and HPV33 to be among the dominant types in bladder tissues in other regions [21,22]. The absence of these types in our cohort may reflect differences in regional prevalence, host genetics, or the impact of HPV vaccination programs in the UK, which primarily target HPV16 and HPV18 [23].

The distribution of HR-HPV genotypes across tumour grades in Table 2 revealed that HPV52 was most commonly detected in Grade 1 tumours, while HPV39 and HPV59 were found only in Grade 3, raising the possibility of genotype-specific associations with tumour aggressiveness. While our data suggest a trend toward increased HPV positivity in Grade 1 tumours, this difference was not statistically significant. Larger studies are required to validate this possible association and to determine whether HPV is more active in early tumourigenesis.

Of the 17 HR-HPV-positive bladder cancers, 6 cases (12.2%) exhibited co-infection with multiple HR-HPV types, as shown in Table 3. Co-infections were most frequent in Grade 1 tumours (19%), compared to Grade 3 (11%) and Grade 2 (7%). Although a direct association between co-infection and tumour grade was not observed, the presence of multiple HR-HPV types suggests potential synergistic interactions that may promote viral persistence or increase oncogenic potential, as is consistent with findings in other HPV-related cancers [7,8].

Specific genotype combinations observed in Table 4, such as HPV16–HPV39, HPV35–HPV66, and HPV16–HPV33–HPV52, further highlight the diversity of co-infections in bladder cancer. These combinations could result in more aggressive oncogenic behaviour, as suggested in cervical cancer literature, although this hypothesis remains to be validated in bladder cancer models [9].

Although DNA-based detection methods confirm the presence of HPV genetic material, they do not differentiate between latent, cleared, or transcriptionally active infections [10,21]. Therefore, this study also assessed the expression of HPV E7 oncoprotein via immunohistochemistry in all HR-HPV-positive samples. Thirteen out of sixteen (81%) samples showed E7 expression, confirming active viral gene expression in the majority of cases. Conversely, three HPV DNA-positive samples were negative for E7 expression, possibly due to early-stage infection or immune-mediated clearance, as is consistent with the “hit-and-run” hypothesis of HPV-related oncogenesis [24]. The ‘hit-and-run’ hypothesis proposes that transient HPV infection may initiate oncogenic transformation but is subsequently lost as tumour cells acquire self-sufficiency [24]. According to this theory, viral DNA or proteins may not be detectable in fully transformed cells, even if the virus triggered initial transformation events.

The variability in E7 staining intensity across samples (Figure 3) may reflect differences in viral load, gene integration status, or host immune responses. Interestingly, some co-infected samples lacked E7 expression, suggesting that not all HR-HPV presence translates into oncogenic activity, which aligns with observations in HPV-mediated oropharyngeal and breast cancers [14].

The absence of HPV E7 protein expression in some HPV DNA-positive samples may be due to the presence of episomal viral forms that have not integrated into the host genome or represent latent infections. These cases likely do not contribute to active oncogenesis and support the importance of distinguishing between transcriptionally active and inactive HPV infection.

Demographic data from this cohort showed that 85% of the bladder cancer patients were male, and the majority were over 60 years of age, matching national and international bladder cancer trends [2,25]. These findings reinforce the representativeness of our sample for the UK bladder cancer population and highlight the need for gender- and age-specific analyses in future studies.

This investigation also has public health implications. The detection of HR-HPV types such as HPV35, HPV52, and HPV66—not currently covered by first-generation HPV vaccines—raises important questions about the scope of current vaccination strategies [23]. If further studies confirm a pathogenic role of these types in bladder cancer, broader or regionally tailored vaccines may be necessary. Moreover, increasing awareness of HPV’s role beyond cervical cancer could improve early detection, prevention, and patient outcomes.

Although HPV DNA was not detected in the four benign bladder tissue specimens in this study, given the small number of benign cases, this absence does not provide sufficient evidence to make any definitive conclusions regarding the role of HPV in benign bladder tissues. Further studies with a larger cohort of benign cases are necessary to better assess the potential presence or absence of HPV in non-cancerous bladder tissue.

Despite the valuable insights provided by this study, there are several limitations that need to be acknowledged. The sample size of 55 bladder tissue specimens is relatively small, which limits the statistical power of our findings and their generalisability. A larger cohort would be beneficial for confirming the prevalence of HR-HPV in bladder cancer and evaluating the potential relationship between specific HPV types and tumour grade or aggressiveness. Additionally, this study is cross-sectional in nature, so it is unable to establish causality or the temporal relationship between HPV infection and bladder cancer development. Longitudinal studies are necessary to further investigate the role of HPV in bladder carcinogenesis over time.

Nevertheless, this is the first UK-based study to investigate the prevalence and expression of 12 HR-HPV genotypes in fresh bladder cancer tissue. The results contribute to a growing body of evidence suggesting that HPV may be involved in bladder carcinogenesis, at least in a subset of cases. These findings suggest the need for further mechanistic studies and larger epidemiological analyses across different populations.

## Figures and Tables

**Table 1:** The histopathological results of patients with suspected bladder cancer at Kingston Hospital. Based on these histopathological results, the most diagnosed bladder cancer cases were transitional cell carcinoma (TCC) cases and squamous cell carcinoma was found to be the rarest cancer type.

	Total Samples N (%)
Total number of samples	55 (100)
Female	8/55 (14.5)
Male	47/55 (85.5)
Age (Year)	
(45–96)	
<60	6/55 (10.9)
61–70	10/55 (18.2)
71–80	18/55 (32.7)
81–90	16/55 (29.1)
>90	5/55 (9.1)
Pathological status	
Squamous cell carcinoma	2/55 (3.6)
Transitional cell carcinoma	49/55 (89.1)
No cancer	4/55 (7.3)

**Table 2:** Prevalence of 12 HR-HPV Types in Bladder Samples. The table demonstrates the prevalence of each of the 12 HR-HPV types in TCC of the bladder. Most prevalent HPV types in cancerous cases were HPV16, 35, and 52 with 5 samples infected.

Pathological Status	HPV Genotypes											
**HPV in TCC n = 17** **17/49 (35%)**	**16**	**31**	**33**	**35**	**18**	**39**	**45**	**59**	**52**	**56**	**58**	**66**
**Grade 1**	2/5 (40)	-	1/1 (100)	2/5 (40)	-	-	1/4 (25)	-	4/5 (80)	-	-	2/2 (100)
**Grade 2**	-	-	-	1/5 (20)	-	-	1/4 (25)	1/2 (50)	-	-	-	-
**Grade 3**	3/5 (60)	-	-	2/5 (40)	-	1/1 (100)	2/4 (50)	1/2 (50)	1/5 (20)	-	-	-
**Total Prevalence of Specific HPV Genotype Cases n =**	5	-	1	5	-	1	4	2	5	-	-	2

**Table 3:** The frequency of HR-HPV infection with multiple HPV genotypes in bladder samples.

Pathological Status	Total Number of Cases	Total HPV + N (%)	HPV Co-Infections N (%)
Transitional Cell Carcinoma Cases	49/49 (100)	17/49 (34.7)	6/49 (12.2)
Grade 1	16	8/16 (50)	3/16 (19)
Grade 2	14	2/14 (14)	1/14 (7)
Grade 3	19	7/19 (37)	2/19 (11)
Total	49	17	6

**Table 4:** HPV co-infection combination types in bladder tissues.

HPV Co-Infection Pairing	Frequency
**HPV16-HPV39**	1
**HPV35-HPV66**	1
**HPV52-HPV66**	1
**HPV45-HPV59**	1
**HPV16-HPV33-HPV52**	1
**HPV35-HPV45-HPV59**	1

## Data Availability

Data is available upon request from the authors.
